# Ultrasmall BiOI Quantum Dots with Efficient Renal Clearance for Enhanced Radiotherapy of Cancer

**DOI:** 10.1002/advs.201902561

**Published:** 2020-01-16

**Authors:** Xin Wang, Zhao Guo, Chenyang Zhang, Shuang Zhu, Lele Li, Zhanjun Gu, Yuliang Zhao

**Affiliations:** ^1^ CAS Key Laboratory for Biomedical Effects of Nanomaterials and Nanosafety Institute of High Energy Physics Chinese Academy of Sciences Beijing 100049 China; ^2^ College of Materials Science and Optoelectronic Technology University of Chinese Academy of Sciences Beijing 100049 China; ^3^ CAS Center for Excellence in Nanoscience National Center for Nanoscience and Technology of China Chinese Academy of Sciences Beijing 100190 China

**Keywords:** intratumoral injection, radiosensitizers, radiotherapy, renal clearance, ultrasmall nanomaterials

## Abstract

Emerging strategies involving nanomaterials with high‐atomic‐number elements have been widely developed for radiotherapy in recent years. However, the concern regarding their potential toxicity caused by long‐term body retention still limits their further application. In this regard, rapidly clearable radiosensitizers are highly desired for practical cancer treatment. Thus, in this work, ultrasmall BiOI quantum dots (QDs) with efficient renal clearance characteristic and strong permeability inside solid tumor are designed to address this issue. Additionally, considering that injection methods have great influence on the biodistribution and radiotherapeutic efficacy of radiosensitizers, two common injection methods including intratumoral injection and intravenous injection are evaluated. The results exhibit that intratumoral injection can maximize the accumulation of radiosensitizers within a tumor compared to intravenous injection and further enhance radiotherapeutic efficacy. Furthermore, the radiosensitizing effect of BiOI QDs is revealed, which is not only attributed to the radiation enhancement of high‐*Z* elements but also is owed to the •OH production via catalyzing overexpressed H_2_O_2_ within a tumor by BiOI QDs under X‐ray irradiation. As a result, this work proposes a treatment paradigm to employ ultrasmall radiosensitizers integrated with local intratumoral injection to realize rapid clearance and high‐efficiency radiosensitization for cancer therapy.

## Graphical abstract

Ultrasmall BiOI quantum dots are developed as efficient radiosensitizers to investigate the potential influence of different injection methods on radiotherapeutic efficacy. Intratumoral administration combined with ultrasmall radiosensitizers can achieve high accumulation in the tumor area, as well as rapid clearance from organisms, leading to enhanced radiotherapeutic efficacy and biosafety for cancer treatment.

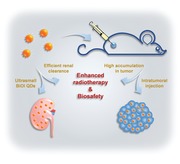

## 1 Introduction

Radiotherapy (RT), using high‐energy ionizing radiation such as γ‐ray or X‐ray for tumor ablation, is one of the most primary methods for cancer treatment.^1^ Despite of its superiority such as the unlimited penetration depth and spacetime‐controlled advantages, the collateral damage to normal tissues induced by the high‐energy ionizing radiation restricts the further development of RT in clinic to a certain extent.^2^ In order to realize better radiotherapeutic efficacy and less unwanted injuries to normal tissues, a variety of nanomaterials, especially those with high‐*Z* elements, have been developed as radiosensitizers to solve the issue, in which these radiosensitizers can enhance the radiotherapeutic efficacy by depositing more radiation dose into tumor even at a low‐dose radiation.^3^ For their further clinical translation, it is highly desirable for these nanoradiosensitizers to be biodegradable or to be eliminated from the body rapidly after treatment so as to avoid long‐term body retention.^4^ However, most nanoparticles can be easily uptaken by the reticuloendothelial system (RES), which may lead to high accumulation in RES organs and tardy clearance, thereby threatening the biosafety to organisms.^5^ In this regard, ultrasmall‐sized inorganic radiosensitizers provide a promising avenue to realize rapid renal clearance for much safer treatments due to their small‐size advantage.^6^ More importantly, ultrasmall nanoparticles can overcome the physiological barriers of tumor imposed by abnormal tumor vasculature and the dense interstitial matrix, exhibiting strong permeability for achieving homogenous distribution of radiosensitizers within solid tumor compared with large‐size nanoparticles.^7^ Although these highlighted merits make ultrasmall nanoparticles more suitable in the nanomaterial‐mediated RT to effectively improve the radiotherapeutic efficacy, there still exist some issues that are nonnegligible points but rarely to be discussed and concerned such as the selection of injection method.

Nowadays, intratumoral injection and intravenous injection are the most common ways adopted in nanomaterial‐mediated tumor radiosensitization.^8^ And each approach has its own advantages. For intravenous injection, it can be theoretically applied to all tumors in situ involving some deep‐seated tumors, in which the nanoradiosensitizers can passively target to tumor sites through the enhanced permeation and retention effect.^9^ However, the portion for these radiosensitizers that can accumulate in tumor sites is usually no more than a quarter of the total injected dose.^10^ And this injection approach may also cause the accumulation of radiosensitizers in healthy tissues, resulting in serious issue of biosafety. For intratumoral injection, it can maximize the content of radiosensitizers within tumor compared to intravenous injection because the strategy can reduce the loss of radiosensitizers during the long‐term circulation in blood, which may achieve better radiotherapeutic efficacy. Nevertheless, the lack of maneuverability for the deep‐seated tumors may limit its practical application. Fortunately, the development of interventional method for delivering drug into deep‐seated tumors may provide new opportunity to overcome the above limitations of intratumoral injection.^11^ It can be seen that the administration methods are crucial to the biosafety of nanoradiosensitizers and radiotherapeutic efficacy, thus deeper research and discussion are important and necessary.

**Scheme 1:**
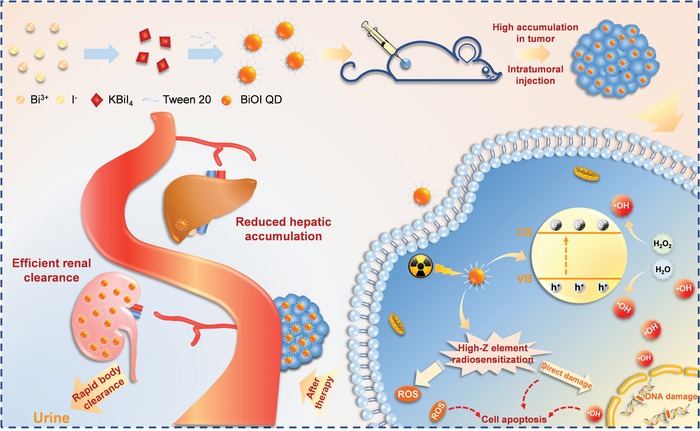
Schematic illustration of the BiOI‐QDs‐based radiotherapeutic progress and biodistribution of BiOI QDs after therapy.

Herein, we developed the ultrasmall BiOI quantum dots (QDs) as radiosensitizers to investigate the potential impact of different injection methods on their biodistribution and radiotherapeutic efficacy. First, the biodistribution experiments are employed to confirm the rapid clearance for these ultrasmall QDs after intratumoral injection and intravenous injection. The results indicate that BiOI QDs can be rapidly eliminated by renal metabolic pathway in any injection method and have a significantly low‐level accumulation in liver and spleen, which forebodes that ultrasmall BiOI QDs can minimize their potential biotoxicity caused by long‐term retention. In addition, the concentration of BiOI QDs inside tumor via intratumoral injection is dramatically higher than that through intravenous injection, which renders the radiosensitizers to give their full play for the best radiotherapeutic outcome when they are injected intratumorally. Intriguingly, the clearance is obviously accelerated in the intratumoral injection group after the X‐ray irradiation. This phenomenon may be ascribed as the destruction of tumor stroma caused by the effectual RT,^12^ which results in the accelerated efflux of nanomaterials. The accumulation of radiosensitizers inside tumor by intratumoral injection before X‐ray treatment and rapid clearance from the organism after the treatment can meet the demands of radiosensitization and biosafety at the same time. Besides, the radiosensitization mechanism of BiOI QDs is also revealed, which attributes to the radiation enhancement of high‐*Z* elements in BiOI QDs and the hydroxyl radical (•OH) production via the catalytic degradation of overexpressed hydrogen peroxide (H_2_O_2_) within tumor by BiOI QDs under X‐ray irradiation. As a result, the work provides a new idea to realize biosafety and high‐efficiency radiosensitization by introducing ultrasmall renal‐clearable nanoparticles as radiosensitizers combined with local intratumoral method, which may contribute to the further clinical translational research (**Scheme** 1).

## 2 Results and Discussion

**Figure 1:**
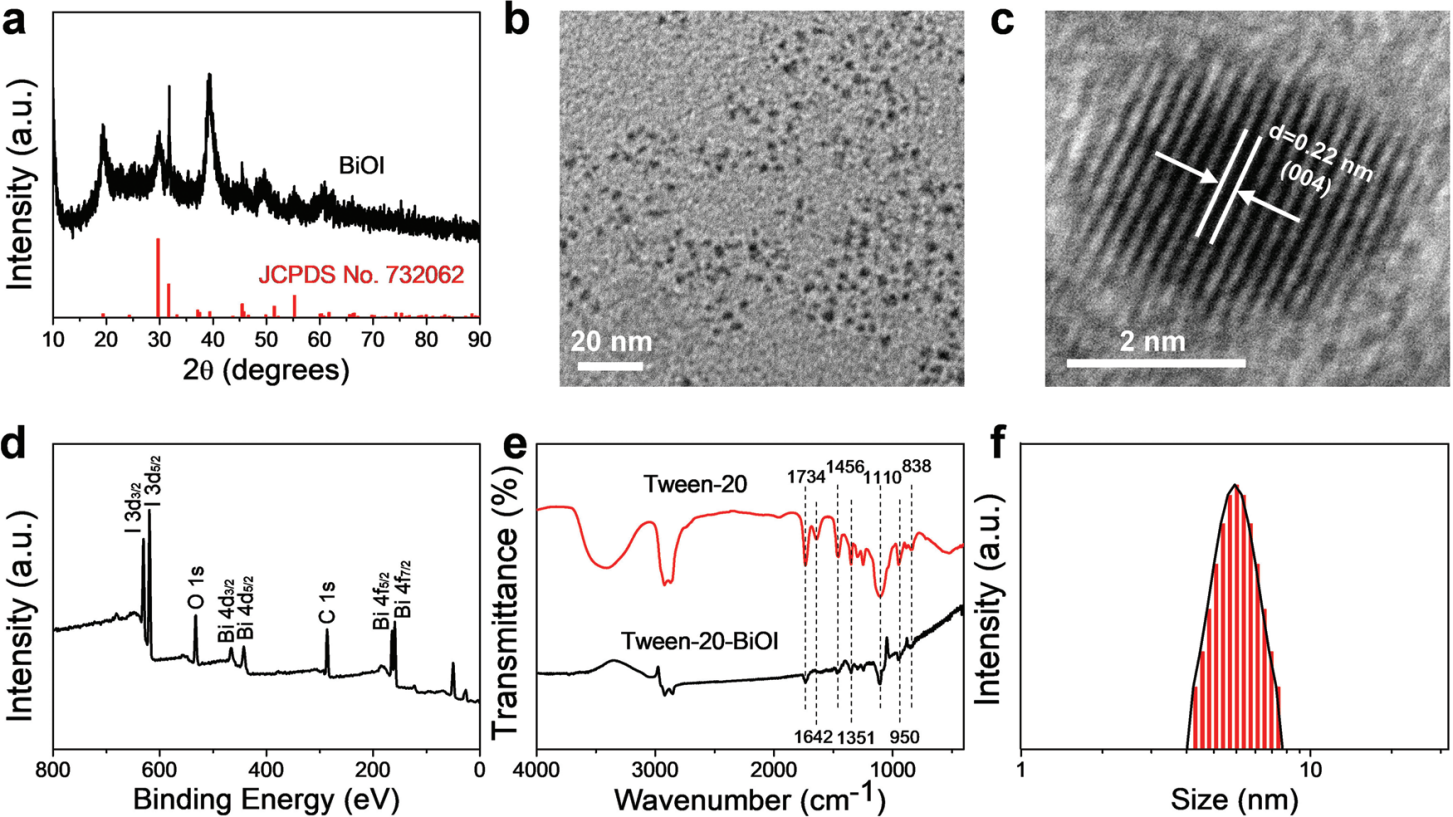
Characterization of the ultrasmall BiOI QDs. a) XRD pattern of BiOI QDs. b) TEM image and c) HRTEM image of BiOI QDs. d) XPS spectra of BiOI QDs. e) FTIR spectrum of BiOI QDs with Tween‐20 modification. f) DLS for the measurement of hydrodynamic size of BiOI QDs.

The ultrasmall BiOI QDs with Tween 20 modification were prepared through a simple two‐step method at room temperature. Typically, all peaks shown in X‐ray powder diffraction patterns (XRD) of the sample match well with tetragonal structure of BiOI (JCPDS 73‐2062; **Figure** 1a). The transmission electron microscopy (TEM) image of the as‐synthesized quantum dots exhibits that the average particle size is around 3 nm (Figure 1b). Additionally, crystal lattice fringes with an interplanar spacing of 0.22 nm can be clearly observed in high‐resolution transmission electron microscope (HRTEM) image (Figure 1c), which is assigned to (004) plane of BiOI. Moreover, energy‐dispersive X‐ray spectroscopy analysis was carried out to confirm the composition of as‐prepared BiOI QDs (Figure S1, Supporting Information), and the chemical state of elements in BiOI QDs was investigated by X‐ray photoelectron spectroscopy (XPS; Figure 1d and Figure S2, Supporting Information). The above results indicated that the high‐quality ultrasmall BiOI QDs were successfully prepared. Furthermore, the Fourier transform infrared (FTIR) spectrum of BiOI QDs was conducted to characterize the surface coating with Tween 20 (Figure 1e). The successful modification with Tween 20 endows BiOI QDs proper zeta potential (−25.47 mV) and good stability in various physiological solutions (Figures S3 and S4, Supporting Information). In addition, the mean hydrodynamic size of BiOI QDs is around 5.51 nm determined by the dynamic light scattering (DLS; Figure 1f), which is smaller than glomerular filtration size threshold (10 nm).^13^ The result indicates that the ultrasmall BiOI QDs have the promising potential for rapid clearance by renal excretion route after the therapy.

**Figure 2:**
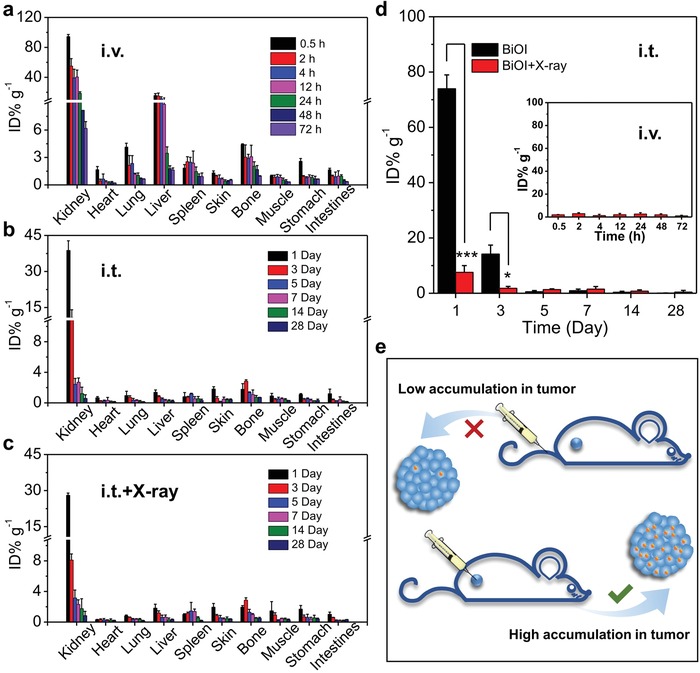
Biodistribution study of BiOI QDs via different administration methods. a) Time‐dependent biodistribution of Bi after intravenous injection (i.v.) of BiOI QDs (500 µg mL^−1^, 200 µL). b) Bi distribution in main organs after intratumoral injection (i.t.) of BiOI QDs (2 mg mL^−1^, 50 µL) without and c) with X‐ray irradiation (6 Gy). d) Biodistribution of Bi in the tumor site via different injection methods. e) Schematic illustration showing the influence of injection method on the distribution of BiOI QDs in tumor site. *p* Values were calculated by the Student's *t* test: *p < 0.05, ***p < 0.001.

Before verifying the BiOI‐QDs‐induced radiosensitizing effect in vitro and in vivo, we investigated the biodistribution of BiOI QDs in the main organs under different injection methods, because it is of significant meaning to biological safety and can also provide valuable guidance for subsequent treatment in vivo. Herein, two common injection methods involving intravenous injection and intratumoral injection were conducted on 4T1‐tumor‐bearing BALB/c mice. And the total injection dose was kept the same in intravenous injection (500 µg mL^−1^, 200 µL) and intratumoral injection (2 mg mL^−1^, 50 µL). First, time‐dependent distribution of Bi was quantified by inductively coupled plasma‐mass spectrometry (ICP‐MS) after intravenous injection of BiOI QDs (**Figure** 2a). It can be found that the hepatic and splenic accumulation is not as high as that of larger nanoparticles in our previous work involving larger BiOI nanoparticles (≈100 nm), in which the larger BiOI nanoparticles showed serious accumulation in liver and spleen.^14^ In this work, the dominating distribution of Bi is detected in kidney, which implies that their excretion pathway in vivo may be mainly through the renal clearance due to their ultrasmall size. Meanwhile, the evident clearance via kidney is also observed in the group of intratumoral injection as illustrated in Figure 2b,c, where the distribution of Bi in kidney (38.78 ID% g^−1^) is much higher than that in liver (1.38 ID% g^−1^) and spleen (0.82 ID% g^−1^) after 1 day post‐injection. And the Bi content in kidney quickly declines over time. Here, it can be found that Bi content in the organs like liver and spleen shows no noticeable accumulation via any injection method, which may be ascribed to the ultrasmall size that enables them to escape from the high uptake by macrophage in RES organs. These results in two injection methods demonstrate that ultrasmall BiOI QDs can ensure the biosafety because of their rapid renal clearance. Indeed, the rapid‐clearance radiosensitizers are ideal for the nowadays fractionated RT in clinic because it requires a very low retention of radiosensitizers in the normal organs before the next irradiation to avoid the irradiation enhancement in the normal organs.^15^ It can be seen that the rapid renal clearance endows ultrasmall nanomaterials containing heavy metal like BiOI QDs with the advantage of reducing the potential adverse effect from long‐term retention, exhibiting great potential for clinical application.

The fact has been proved that BiOI QDs can reach the kidney for rapid clearance no matter through intravenous or intratumoral administration. However, for different ways of injection, it shows a significant influence on the distribution of radiosensitizers in tumor. As illustrated in Figure 2d, the Bi content in the tumor of mice via intravenous injection method is never more than 3 ID% g^−1^ during the inspection time. Whereas, an obvious difference occurs when it comes to intratumoral injection, in which the content of Bi in tumor is around 73.96 ID% g^−1^ at 1 day post‐injection and it is speculated to be higher when the injection is just accomplished. Specifically, it is worthy to be mentioned that the clearance is obviously accelerated in the intratumoral injection group with the X‐ray irradiation compared to that without X‐ray irradiation within the same time frame (i.e., from 73.96 to 7.58 ID% g^−1^ at 1 day after injection). That is to say, the intratumoral injection is beneficial to the accumulation of radiosensitizers within tumor to improve the radiotherapeutic efficacy, and the succedent X‐ray irradiation not only realizes the therapy of tumor but also greatly accelerates the elimination of the nanomaterials from the organism after the therapeutic process. It may be attributed to the strong destruction of tumor stroma caused by high‐energy X‐ray during RT, and thus the nanoradiosensitizers run off from the tumor after the completion of their mission to preclude long‐term retention and potential toxicity. According to the results of the biodistribution in the main organs and tumor, two injection methods exhibit minor differences in the rapid clearance of BiOI QDs by kidney, while the accumulation of BiOI QDs in tumor by intratumoral injection is apparently higher than that by intravenous injection and this distinction is illustrated in Figure 2e. Therefore, for the ultrasmall BiOI QDs, intratumoral injection is the optimal injection method to achieve their maximum effective utilization in RT. Taken together, the ultrasmall radiosensitizers combined with intratumoral administration not only ensure the high accumulation in tumor area but also lead to rapid clearance from the organisms, making them obvious superiority in effective cancer treatment and biosafety.

**Figure 3:**
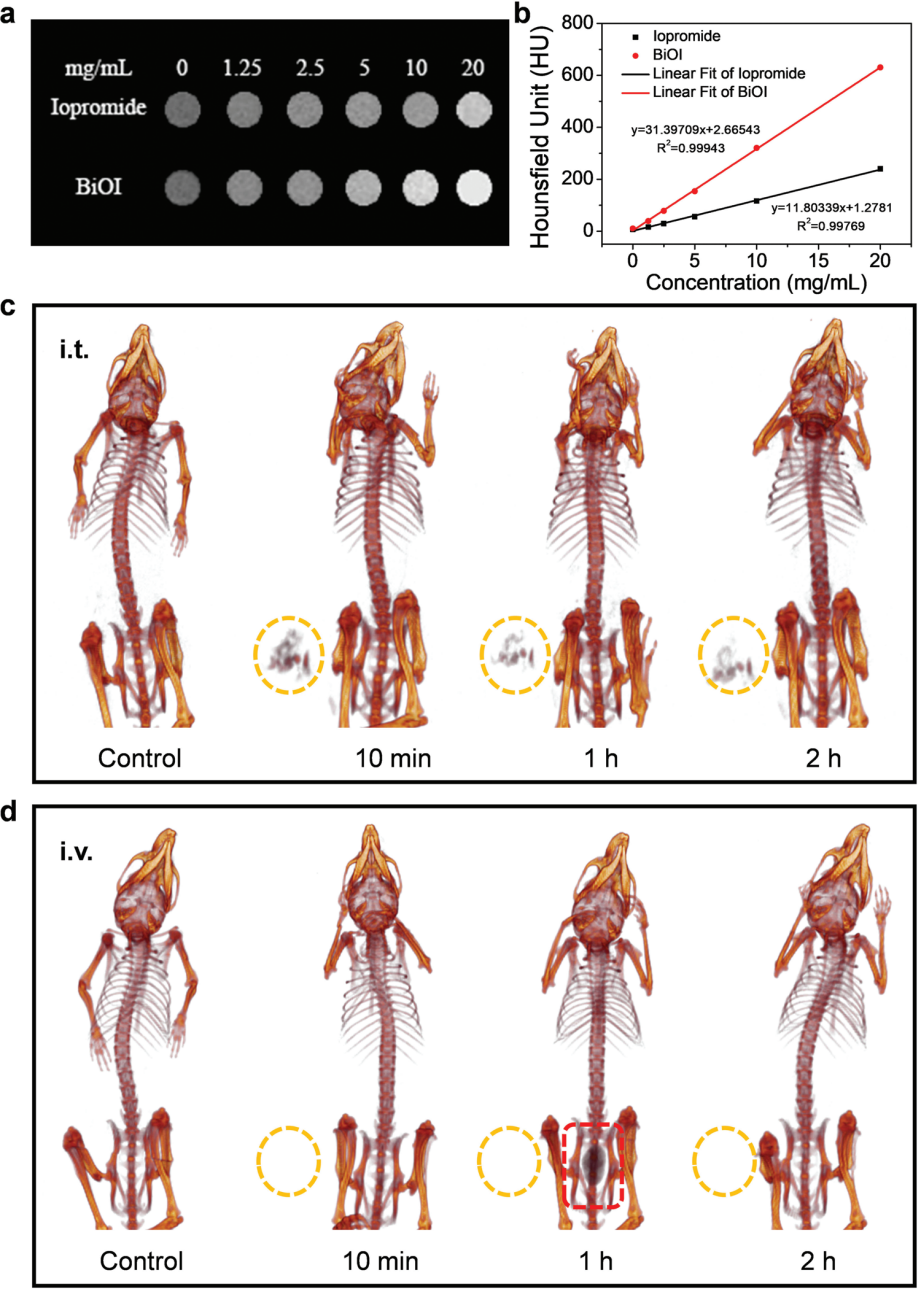
CT imaging assessment of BiOI QDs. a) CT images of BiOI QDs and iopromide in vitro at various concentrations. b) Corresponding CT value of BiOI QDs and iopromide. c) In vivo CT images taken at different time after intratumoral injection of BiOI QDs. d) In vivo CT images of a tumor‐bearing mouse after intravenous injection of BiOI QDs at various time points.

In addition to ICP‐MS analysis of the BiOI QDs distribution in the main organs and tumor, the BiOI QDs‐mediated X‐ray computed tomography (CT) imaging was also employed to further visually testify the advantage of intratumoral injection. By comparing with iopromide (a commercial clinical agent), we first assessed the CT imaging potential of BiOI QDs in vitro due to their strong X‐ray attenuation. We found that BiOI QDs exhibited stronger CT signal intensity (630 HU) than that of iopromide (240 HU) under the same concentration of 20 mg mL^−1^ (**Figure** 3a,b). Then 4T1‐tumor‐bearing BALB/c mice were used for assessing CT imaging ability under different injection methods in vivo. For the group of intratumoral injection, a strong CT contrast signal was observed in tumor site after the injection, which verifies the CT imaging capability of BiOI QDs as well as the effective tumor accumulation of radiosensitizers through intratumoral injection (Figure 3c). Moreover, after 2 h post‐injection by intratumoral injection, the tendency of rapid clearance of BiOI QDs can be also observed through the weakened signal in tumor site. By contrast, no visible signal can be observed in tumor area after intravenous injection of BiOI QDs, indicating the lower accumulation of BiOI QDs in tumor. The similarity of these two injection methods is that no noticeable CT signals emerge in liver and spleen no matter through intratumoral injection or intravenous injection, further testifying that ultrasmall size enables BiOI QDs to escape from the high uptake by macrophage in RES organs.^16^ In addition, the clear CT signal appears in bladder after 1 h post‐injection by intravenous injection (Figure 3d), demonstrating that the BiOI QDs can be eliminated through the kidney into urine. All these CT imaging results are well consistent with the aforementioned biodistribution experiments by ICP‐MS analysis, which further demonstrates that both the two injection methods cannot cause accumulation of BiOI QDs in RES tissues and intratumoral injection may be the optimal choice to effectively realize the accumulation of ultrasmall sensitizers into tumor for safe and high‐efficiency radiosensitization.

**Figure 4:**
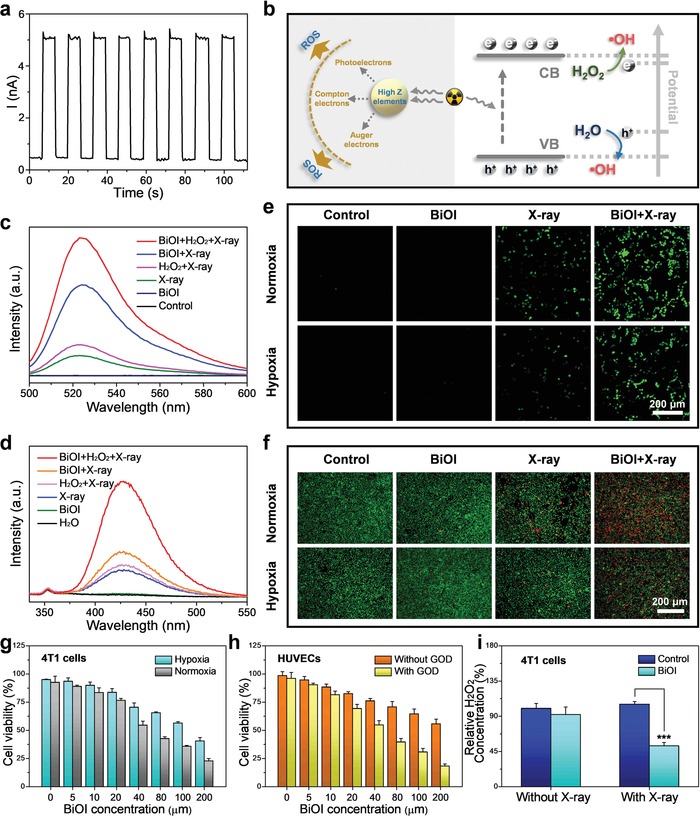
Radiosensitizing effect study of BiOI QDs. a) Photocurrent response of BiOI QDs to X‐ray. b) A plausible radiosensitizating mechanism illustration based on BiOI QDs. c) Evaluation of ROS generation ability of BiOI QDs under various treatments. d) Fluorescence spectra for •OH generation measurement with different treatments. e) Confocal images of intracellular ROS generation in 4T1 cells under normoxic and hypoxic condition. f) Live–dead staining assay conducted in 4T1 cells under normoxic and hypoxic condition. g) CCK‐8 assay of 4T1 cells incubated with BiOI QDs under X‐ray at normoxic and hypoxic condition. h) Cell viability of HUVECs incubated with BiOI QDs under X‐ray with or without GOD. i) Detection of H_2_O_2_ concentration in 4T1 cells after various treatments. *p* Values were calculated by the Student's *t* test: ***p < 0.001.

After exploring the selection of injection method for ultrasmall BiOI QDs, the potential of BiOI QDs as radiosensitizers was systematically evaluated. Due to the presence of high‐*Z* element bismuth (*Z* = 83) and iodine (*Z* = 53) with strong X‐ray absorption, the BiOI QDs could efficiently deposit more X‐ray energy within tumor and cause enhanced damages to tumor. Additionally, BiOI QDs as typical semiconductor photocatalytic materials with the inherent superiority to utilize their energy band structure to generate reactive oxygen species (ROS), may further enhance the radiotherapeutic efficacy by X‐ray‐induced catalytic reaction, achieving the controllable treatment of tumor. According to their energy band structure, they have the potential to catalyze H_2_O_2_ into highly cytotoxic •OH under X‐ray irradiation. Fortunately, the H_2_O_2_ are over‐produced in the tumor microenvironment (TME) but not in normal tissues.^17^ Therefore, the ultrasmall semiconductors photocatalytic nanomaterials under X‐ray irradiation may provide a promising strategy to realize enhanced therapeutic efficacy to tumor while not in normal tissues. Herein, a plausible mechanism is proposed (**Figure** 4b). In detail, the X‐ray‐triggered electrons and holes reach to conduction band and valence band with reduction and oxidation property, respectively. Then, H_2_O_2_ as the electron acceptor can be reduced into •OH, while H_2_O as hole acceptor will be oxidized to •OH based on proper potential location.^18^ Based on this, the X‐ray‐triggered catalytic performance of BiOI QDs was assessed. First, the X‐ray‐triggered electron–hole pair generation was validated by detecting photocurrent response of BiOI QDs to X‐ray, where the photocurrent showed immediate increase for each switch‐on of X‐ray (Figure 4a). Next, 2′,7′‐dichlorofluorescein (DCFH‐DA) was employed to identify the production of ROS (Figure 4c), the presence of BiOI QDs leads to 4.48‐fold augment in fluorescent intensity than radiosensitizer‐absent group under X‐ray irradiation. Then, the addition of H_2_O_2_ further improves ROS generation by half, which may be ascribed to more •OH generation induced by BiOI‐based catalytic reaction under X‐ray irradiation. To verify that the enhancement in ROS mostly comes from •OH after the addition of H_2_O_2_, terephthalate (TA) was employed for •OH detection. As shown in Figure 4d, the fluorescent intensity of •OH in the presence of BiOI QDs and H_2_O_2_ under X‐ray irradiation is 2.53‐fold higher than the group treated only with BiOI QDs under X‐ray irradiation, elucidating the BiOI QDs can effectively catalyze H_2_O_2_ into •OH for enhanced RT.

Sparked by their effective generation of ROS, we next evaluated the ROS generating ability of BiOI QDs at cellular level. Before that, the cytotoxicity of BiOI QDs was assessed via the Cell Counting Kit 8 (CCK‐8) assay, in which no obvious toxicity was induced by BiOI QDs in murine breast carcinoma cells (4T1 cells) (Figure S5, Supporting Information). Then, DCFH‐DA was applied to monitor the intracellular ROS level. As displayed in Figure 4e, the brightest fluorescence signal can be observed in normoxic 4T1 cells treated with both BiOI QDs and X‐ray, indicating that BiOI QDs with X‐ray irradiation have the potential to kill tumor cells via ROS enhancement. More importantly, according to previous studies in catalytic performance of BiOI QDs, the enhanced ROS mainly comes from the •OH generation in the presence of H_2_O_2_ without reliance on oxygen. The results demonstrate that BiOI QDs may still generate considerable ROS via catalyzing overexpressed H_2_O_2_ in hypoxic 4T1 cells for enhanced radiotherapeutic efficacy. Therefore, the ROS generation under hypoxic 4T1 cells was also evaluated (Figure 4e), in which the significative fluorescence signal in the group of BiOI QDs with X‐ray irradiation indicates that BiOI QDs may be used to address hypoxia‐tumor RT.

To confirm that BiOI QDs have the enhanced radiotherapeutic efficacy under hypoxic condition, a series of killing effect experiments were evaluated. First, the live–dead staining assay was carried out to visually verify the tumor cell‐killing capacity of BiOI QDs (Figure 4f), in which the BiOI QDs with X‐ray irradiation result in the most obvious killing effect to normoxic and hypoxic 4T1 cells. Besides, the CCK‐8 assay was further used to confirm the killing effect of BiOI QDs under X‐ray irradiation (Figure 4g), which is consistent with the results of live–dead staining assay. All of above results imply that BiOI QDs have great potential in tumor RT. Moreover, due to that the H_2_O_2_ is overexpressed in cancer cells but not in normal cells, their decomposition into •OH triggered by X‐ray can only happen in tumor cells rather than normal cells, which signifies that BiOI QDs possess the ability to selectively enhance the killing effect to tumor. Therefore, the cell viability of normal cells (human umbilical vein endothelial cells, HUVECs) incubated with BiOI QDs under X‐ray irradiation was evaluated (Figure 4h). From the results, the cell viability of HUVECs incubated with BiOI QDs under X‐ray irradiation only showed moderate decrease, which indicates the BiOI QDs with X‐ray irradiation fail to produce abundant •OH in HUVECs with low‐level H_2_O_2_. It can be seen that H_2_O_2_ plays an important role in enhancing the radiosensitizing effect of BiOI QDs. To certify this, HUVECs were treated with glucose oxidase (GOD) to elevate H_2_O_2_ content: then, the cell viability of HUVECs with elevated H_2_O_2_ declines to 18.2%. Similarly, in order to prove the role of H_2_O_2_ in tumor cells, we detected the change of H_2_O_2_ concentration in 4T1 cells and human cervical cancer cells (HeLa cells) after different treatments, respectively. The obvious decrease of H_2_O_2_ in the group incubated with BiOI QDs under X‐ray was detected in both 4T1 cells (Figure 4i) and HeLa cells (Figure S6, Supporting Information), which verified that the intracellular H_2_O_2_ was indeed decomposed by BiOI QDs under X‐ray irradiation. All these results demonstrate that BiOI QDs have the potential to enhance the radiotherapeutic effect via promoting intracellular ROS generation through high‐*Z* element radiosensitization and X‐ray‐induced •OH generation.

**Figure 5:**
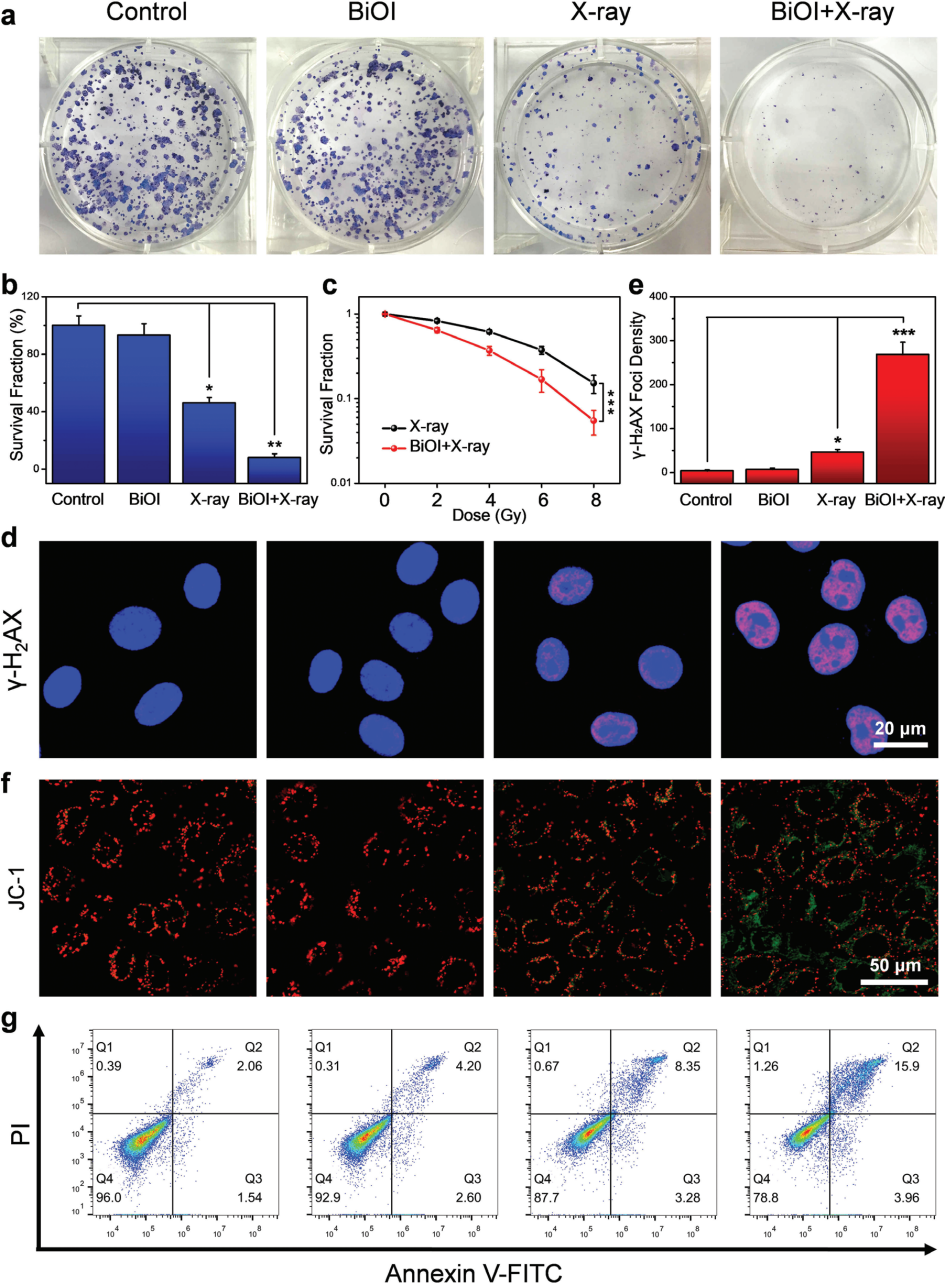
Enhanced RT of BiOI QDs in vitro. a) Colony formation of 4T1 cells treated with BiOI QDs (50 µg mL^−1^) and X‐ray irradiation (6 Gy). b) Survival fraction from colony assay in each group. c) Dose‐effect curve from colony assay in 4T1 cells under X‐ray with or without BiOI QDs. d) Confocal images of DNA damage in 4T1 cells treated with BiOI QDs (50 µg mL^−1^) and X‐ray irradiation (6 Gy). e) Normalized fluorescence spot number of γ‐H_2_AX staining 4T1 cells after various treatments. f) JC‐1 staining in 4T1 cells to detect the change in mitochondrial membrane potential. g) Apoptosis/necrosis detection of 4T1 cells via flow cytometry analysis. *p* Values were calculated by the Student's *t* test: *p < 0.05, **p < 0.01, ***p < 0.001.

The above results in vitro have indicated that BiOI QDs under X‐ray irradiation possess enhanced tumor‐killing capacity via X‐ray‐triggered ROS generation. To further evaluate the radiotherapeutic efficacy, the clonogenic assay was carried out to visually observe the radiosensitization effect of BiOI QDs. The colony formation of 4T1 cells incubated with BiOI QDs showed no significant reduction compared with the control group, but it drastically declined to 8.01% when 4T1 cells were treated with BiOI QDs under X‐ray irradiation (**Figure** 5a,b). And the sensitizer enhancement ratio of BiOI QDs with X‐ray irradiation was calculated to be 1.4 based on the clonogenic assay (Figure 5c). The results demonstrate that BiOI QDs under X‐ray irradiation can effectively inhibit tumor cell proliferation. Considering that the enhanced radiotherapeutic efficacy may be attributed to the oxidative damage of ROS to DNA generated by BiOI QDs under X‐ray irradiation,^19^ the damage of the DNA double‐strand was evaluated via γ‐H_2_AX detection in 4T1 cells. Compared with the group treated with X‐ray alone, obvious γ‐H_2_AX foci density is observed in the 4T1 cells treated with BiOI QDs under X‐ray irradiation, exhibiting about 5.87‐fold enhancement (Figure 5d,e). Next, in order to make clear the death mechanism of 4T1 cells after different treatments, the change of mitochondrial membrane potential was revealed by JC‐1 staining.^20^ The decreasing red fluorescence and increasing green fluorescence observed in 4T1 cells treated with BiOI QDs and X‐ray irradiation implied the obviously disturbed mitochondrial membrane potential, suggesting that BiOI QDs with X‐ray irradiation may result in more serious cell apoptosis effect to 4T1 cells (Figure 5f). This speculation was certified through Annexin V‐FITC (AV)/propidium iodide (PI) apoptosis assay. It can be seen that the late apoptosis cells in the group treated with BiOI QDs and X‐ray irradiation are higher than those in the group treated with X‐ray alone (Figure 5g). Moreover, in order to further ascertain the radiosensitizing ability of BiOI QDs, classical experiments were also conducted on HeLa cells and efficient radiotherapeutic outcome was observed (Figure S7, Supporting Information). These results in different tumor cell lines may indicate that BiOI QDs have universality to realize enhanced tumor RT via X‐ray‐triggered ROS generation.

**Figure 6:**
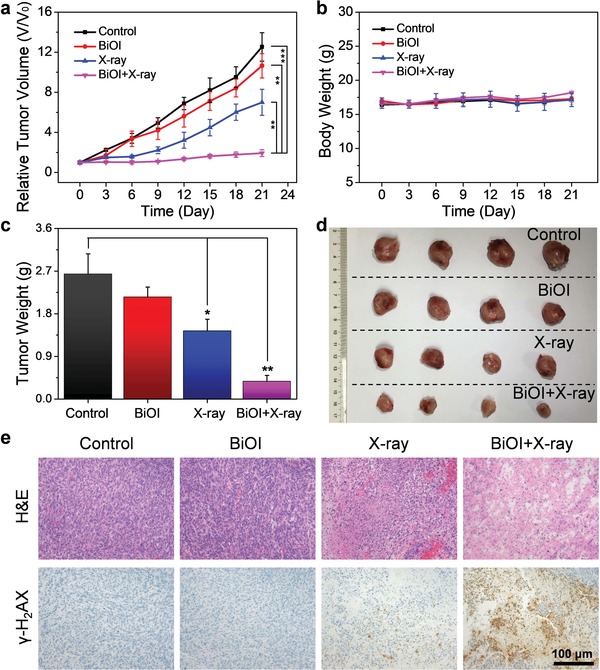
BiOI QDs sensitized RT in vivo. a) Relative tumor volume profile of different groups: Control, BiOI, X‐ray, BiOI + X‐ray. b) Body weights of 4T1‐tumor‐bearing mice after treatments. c) Weights of excised tumors of each group at 21st day after treatments. d) Photograph of excised tumors in each group at 21st day after treatments. e) Tumor images with H&E and γ‐H_2_AX staining after treatments. *p* Values were calculated by the Student's *t* test: *p < 0.05, **p < 0.01, ***p < 0.001.

Then, encouraged by high‐efficiency radiotherapeutic efficacy in vitro, BiOI QDs may exhibit considerable antitumor effect in vivo. Therefore, BALB/c mice bearing 4T1 tumor were randomized into four groups (control, BiOI, X‐ray, BiOI + X‐ray) to verify the feasibility of BiOI QDs as radiosensitizers for enhanced RT in vivo. Based on the results of CT imaging and biodistribution analysis, we chose the intratumoral injection for the administration of BiOI QDs to maximize their accumulation in tumor site and reduce side effects in other organs. After intratumoral injection with phosphate‐buffered saline (PBS) or BiOI QDs, the RT (dose = 6 Gy) was conducted close behind. Then, tumor volume of each mouse was monitored every 3 days during the experimental period (**Figure** 6a). It can be seen that RT alone cannot inhibit the tumor growth effectively, while evident antitumor efficacy is observed in the group treated with BiOI QDs and X‐ray irradiation with the final relative tumor volume 84.30% lower than that of the control group. Moreover, tumor weight and photographs were obtained at the 21st day after treatment, which were consistent with the result of tumor volume (Figure 6c,d). To further evaluate the radiosensitizing effect of BiOI QDs, the pathology change in tumor was revealed (Figure 6e), and the most severe destruction to tumor cells was observed in the group treated with BiOI QDs and X‐ray irradiation from hematoxylin and eosin (H&E) staining. Additionally, aggravated damage to DNA of tumor cells was found in the group treated with BiOI QDs and X‐ray through γ‐H_2_AX stained tumor slices, which is consistent with our in vitro results. These results demonstrate that BiOI QDs as efficient radiosensitizers can significantly improve radiotherapeutic efficacy to cancer cells, especially together with the intratumoral injection for bringing about localized and maximized damage to tumor. Moreover, to confirm the result, the investigation was conducted on another tumor xenograft (HeLa cervical cancer model) and analogous radiotherapeutic outcome was obtained under the same protocol (Figure S11, Supporting Information). All the results demonstrate that the combination of intratumoral injection and RT sensitized by ultrasmall BiOI QDs leads to an effective therapeutic outcome, revealing the advantage of topical administration. Thus, with the development of interventional therapy, it is a promising strategy to rationally integrate the interventional method and nanomaterial‐sensitized RT to achieve a more tumor‐focused therapy for enhanced therapeutic efficacy to cancer treatment.

Furthermore, the body weights of mice showed no sharp fluctuation after treatment, suggesting that BiOI QDs did not induce noticeable systemic toxicity (Figure 6b). Histopathological study of major organs was also evaluated through H&E staining and no obvious organ abnormality was observed (Figures S8 and S12, Supporting Information). Devoid evidence of inflammation caused by possible adverse effect was found according to the blood routine examination of mice (Figures S9 and S13, Supporting Information). What is more, blood biochemistry assessment was carried out to assess the liver function through alanine aminotransferase (ALT) and aspartate transaminase (AST), meanwhile the kidney function was evaluated via urea nitrogen (UREA) and creatinine (CREA), and no distinct difference was found compared to untreated group (Figures S10 and S14, Supporting Information). All these assessments conducted in two in vivo model (4T1 mammary cancer model and HeLa cervical cancer model) point to no long‐term toxicity of BiOI QDs, indicating their promising bio‐application potential.

## 3 Conclusion

In summary, we developed the ultrasmall BiOI QDs as efficient radiosensitizers and investigated the potential influence on the radiotherapeutic efficacy through different injection methods. The results of biodistribution indicate that the ultrasmall radiosensitizers combined with intratumoral administration not only ensure the high accumulation in tumor area compared with intravenous injection but also lead to rapid clearance from the organisms, making them obvious superiority in effective cancer treatment and biosafety. Moreover, the highly cytotoxic •OH generation in response to the particular TME via X‐ray‐activated catalytic reaction could selectively and efficiently intensify the lethality of BiOI QDs to tumor with X‐ray irradiation. Overall, this work proposes a treatment paradigm that not only realizes the direct intratumoral‐delivery of radiosensitizers by topical intratumoral administration, but also achieves rapid body clearance and reduced potential side effects through employing the ultrasmall radiosensitizers featured with efficient renal excretion, meeting the demands of radiosensitization and biosafety for further clinic translation in cancer treatment.

## 4 Experimental Section

##### Materials

Bismuth nitrate pentahydrate (Bi(NO_3_)_3_·5H_2_O, 98%) was purchased from Alfa Aesar Ltd. Potassium iodide (KI, 99%) was obtained from Sinopharm Chemical reagent Co., Ltd. TA and Tween 20 were purchased from Sigma‐Aldrich. Ethanol was provided by the Beijing Chemical Reagent Company. Hydrogen Peroxide Assay Kit, Reactive Oxygen Species Assay Kit, mitochondrial membrane potential assay kit with JC‐1 (JC‐1), Hoechst 33342 and Annexin V‐FITC Apoptosis Detection Kit were purchased from Beyotime Institute of Biotechnology. RPMI‐1640 and Dulbecco's modified Eagle medium (DMEM) were supplied by HyClone Company, USA. Fetal bovine serum (FBS) and penicillin/streptomycin were obtained from Gibco, Shanghai, China. CCK‐8 and γ‐H_2_AX antibodies were bought from Dojindo Laboratories in Japan and Cell Signaling Technology Company in USA, respectively. Iopromide was purchased from Bayer AG. All chemicals were used as received. Deionized (DI) water (18 MΩ cm) was used throughout the process.

##### Synthesis of BiOI QDs

In a typical synthesis procedure, KI (5 mmol) was dissolved in the mixture of DI water (3 mL) and ethanol (5 mL). Then Bi(NO_3_)_3_·5H_2_O (1 mmol) was added to this solution under ultrasonication. After centrifugation at 12 000 r min^−1^ for 3 min, the supernatant was injected to 1% Tween 20 aqueous solution. After another 3 min centrifugation (8000 r min^−1^), the precipitate was removed and the supernatant was further treated with ultrafiltration (MWCO 50 kDa, Millipore) to remove the excess ions and Tween 20. Then, the aqueous solution of BiOI QDs was obtained.

##### Characterization

The crystallography of BiOI QDs was determined by the powder XRD using a Bruker D8 Advance X‐ray diffractometer with Cu‐K_α_ radiation (λ = 1.5406 Å). TEM images were obtained by Tecnai G2 20 S‐TWIN. The states of elements were characterized by XPS with spectrometer (ESCALab250i‐XL) using the monochromatic Al‐K_α_ radiation (1486.6 eV). Elemental species were analyzed by energy‐dispersive X‐ray spectroscopy. Measurement of the size distribution and Zeta potential was carried out through Nano‐ZS90 (Malvern). FT‐IR spectra of the samples were recorded on a Fourier Transform Infrared Spectroscopy (Nicolet iN10, Thermo Fisher).

##### ROS Detection

Fluorescent probe DCFH‐DA was employed for the ROS detection. First, the chemical hydrolyzation was conducted before detection. Briefly, under dark condition, DCFH‐DA in dimethyl sulfoxide reacted with NaOH (0.01 m), then phosphate buffer (25 × 10^−3^ m, pH = 7.2) was used to stop the reaction after 30 min. Six groups (control, BiOI QDs, X‐ray, BiOI QDs+X‐ray, H_2_O_2_+X‐ray, and BiOI QDs+H_2_O_2_+X‐ray) were set. The dose of ROS probe, H_2_O_2_, BiOI QDs, and X‐ray irradiation were 10 × 10^−6^ m, 100 × 10^−6^ m, 50 µg mL^−1^, and 6 Gy, respectively. Then the ROS generation was detected by fluorescence spectroscopy (Fluorolog‐3, Horiba, Ltd.), and the excited wavelength was 488 nm.

##### Hydroxyl Radical (•OH) Detection

For the detection of •OH, TA was added to each group with the concentration of 50 µg mL^−1^. And the concentration of H_2_O_2_ and BiOI QDs were 100 × 10^−6^ m and 50 µg mL^−1^. Then X‐ray irradiation of 6 Gy was conducted. Next, the aqueous solution of each group was detected by fluorescence spectroscopy (excited wavelength: 315 nm).

##### Cell Culture

HUVECs, 4T1 cells, and HeLa cells were cultured in a humidified atmosphere with 5% CO_2_ at 37 °C. The 4T1 and HeLa cells were maintained in the RPMI‐1640 culture medium containing 10% FBS and 1% penicillin/streptomycin. HUVECs were cultured with similar medium ratio and PRMI‐1640 was replaced by DMEM culture medium.

##### Live–Dead Staining Assay

4T1 cells were seeded into 24‐well plates. After attachment, 50 µg mL^−1^ BiOI QDs were added to the cells for 24 h and then irradiated with X‐ray (6 Gy). Hypoxic condition was simulated via cobalt chloride (CoCl_2_, 100 × 10^−6^ m) in culture medium. After removing the medium, the live cells and dead cells were dyed with calcein AM and PI solution, respectively. Then, the cells were observed by inverted luminescence microscope (Olympus X‐73, Japan).

##### In Vitro Cytotoxicity Study

4T1 cells, HeLa cells, and HUVECs were seeded in 96‐well cell culture plates for 24 h, respectively. Then the culture medium was replaced by medium with increasing concentrations of BiOI QDs (0, 5, 10, 20, 40, 80, 100, and 200 µg mL^−1^) for further incubation. CoCl_2_ of 100 × 10^−6^ m and GOD of 0.01 U mL^−1^ were used to simulate hypoxic condition and regulate intracellular H_2_O_2_ when necessary. After 24 h, the cells were washed and maintained with 10% CCK‐8 reagent in RPMI‐1640 or DMEM for 1 h. At last, the cell viability was evaluated through detecting absorbance in 450 nm via a microplate reader (Thermo Scientific, Multiskasn MK3).

##### Intracellular Hydrogen Peroxide Detection

4T1 cells were seeded into 6‐well plates. After 24 h, the cells were co‐incubated with 50 µg mL^−1^ BiOI QDs for another 24 h and then irradiated with X‐ray (6 Gy). Next, after wash and trypsinization, the intracellular H_2_O_2_ was detected by Hydrogen Peroxide Assay Kit.

##### Detection of ROS Generation In Vitro

Both 4T1 cells and HeLa cells were incubated in confocal dishes, respectively. After attachment, 50 µg mL^−1^ BiOI QDs were added to the cells for 24 h. Hypoxic condition was simulated via 100 × 10^−6^ m CoCl_2_ in culture medium. The cells were washed with PBS and co‐incubated in 1 mL PBS with 10 × 10^−6^ m DCFH‐DA and 1 µg mL^−1^ Hoechst 33342 in the dark for 30 min. Afterward, the cells were washed and irradiated under X‐ray (6 Gy) in fresh culture medium. Finally, the signal was captured with the confocal fluorescence microscopy (Nikon A1).

##### In Vitro Clonogenic Assay

The cells were seeded into 6‐well plates with various density (125, 250, 500, 1000, and 2000 cells per well) and allowed to attach at 37 °C. Afterward, the culture medium was replaced by 50 µg mL^−1^ BiOI QDs for another 24 h and then the cells were exposed with X‐ray irradiation of different dosages (0, 2, 4, 6, and 8 Gy). Then all cells were washed and maintained with culture medium at 37 °C for a week. Finally, 4% paraformaldehyde and Giemsa dye were used for fixation and staining of cells, respectively. The survival fraction counting of the colonies was carried out to evaluate the effects of different treatments.

##### DNA Damage Evaluation In Vitro

4T1 and HeLa cells were divided into four groups (control, BiOI QDs, X‐ray, BiOI QDs+X‐ray), respectively. 3 h after different treatments, the cells were maintained in paraformaldehyde (4%) and Triton‐X 100 (0.2%) to be fixed and permeated, respectively. Then, the PBS solution containing 5% FBS and 1% Triton X‐100 was employed as blocking buffer to treat the cells. 1 h later, the blocking buffer was replaced by anti‐phospho‐histone γ‐H_2_AX rabbit monoclonal antibody (dilution 1:1000) at 4 °C. After 12 h, the cells were rinsed with PBS, following by another 1 h incubation with sheep anti‐rabbit secondary antibody (dilution 1:500). After another rinse with PBS, the cells were stained with Hoechst 33342 to visualize cell nuclei and observed by confocal fluorescence microscopy (Nikon A1).

##### Apoptosis Assay

The cells were maintained in 6‐well culture plates and allowed to attach at 37 °C. After 24 h, the cells were treated with BiOI QDs (50 µg mL^−1^) for 24 h. Next, the cells were rinsed and exposed to X‐ray irradiation (6 Gy). 24 h later, the cells were trypsinized and rinsed. At last, Annexin V‐FITC/PI kit was used to dye the cells, and flow cytometry (Accuric6, BD, USA) was used to analyze cell apoptosis.

##### JC‐1 Assay

4T1 cells were seeded in confocal dishes (5 × 10^4^ per dish) and divided into four groups (control, BiOI QDs, X‐ray, BiOI QDs+X‐ray). Then the cells were incubated with 50 µg mL^−1^ BiOI QDs. After 24 h, the cells in corresponding groups were irradiated to X‐ray (6 Gy). Next, the cells were dyed with JC‐1 and then observed with confocal fluorescence microscopy (Nikon A1).

##### RT with BiOI QDs In Vivo

The animal experiments were under protocols approved by the Key Laboratory for Biomedical Effects of Nanomaterials and Nanosafety (Institute of High Energy Physics, CAS). The 4T1 tumor models and HeLa tumor models were developed by subcutaneous injection. Briefly, 1.0 × 10^6^ 4T1 cells or HeLa cells suspended in 100 µL PBS were subcutaneously injected to the right hind legs of BALB/c female mice or female nude mice (6 weeks old), respectively. Both 4T1‐tumor‐bearing mice and HeLa‐tumor‐bearing nude mice were randomly randomized into four groups (a) control, b) BiOI, c) X‐ray, d) BiOI+X‐ray) with four mice in each group. The mice in each group were intratumorally injected with 25 µL PBS or BiOI QDs (2 mg mL^−1^), respectively. Then X‐ray radiation of 6 Gy was conducted upon the tumors in groups (c) and (d). After different disposes, the mice were fed with sterilized water and food, meanwhile, the tumor growth and mouse weight were monitored closely. A formula was used to calculate tumor volume: volume = (length × width^2^)/2. After 21 days of treatment, every mouse was sacrificed and all the tumors were weighted. Then the heart, liver, spleen, lung, kidney, and tumor of all the mice were excised for histological evaluation. The blood sample of each mouse was collected for blood hematology and biochemistry analyses at the animal department of Peking University medical laboratory.

##### Histology Analysis In Vivo

The major organs (heart, liver, spleen, lung, kidney) and tumors were fixed and embedded into paraffin, then they were sliced and stained with H&E or γ‐H_2_AX antibody. The inverted luminescence microscope (Olympus X‐73, Japan) was used to obtain microscopy images of these slices.

##### CT Imaging

Various concentrations of BiOI QDs and iopromide were dissolved in 0.5% agarose gel for in vitro CT imaging by the Quantum GX microCT Imaging System (PerkinElmer, Inc.). For CT imaging in vivo, 4T1‐tumor‐bearing BALB/c mice were scanned at different time (10 min, 1 h, 2 h) after intravenous injection and intratumoral injection with BiOI QDs, respectively.

##### Biodistribution of BiOI QDs

The time‐dependent biodistribution of BiOI QDs in major organs or tissues, including the heart, liver, spleen, lung, kidney, stomach, intestine, skin, bone, muscle, and tumor was measured by ICP‐MS (Thermal Elemental X7, Thermal Fisher Scientific Inc., USA) after complete digestion in HNO_3_/H_2_O_2_ (2:1, v/v) at around 220 °C. The mice were sacrificed and the major organs or tissues were collected at the time of 0.5, 2, 4, 12, 24, 48, and 72 h after intravenous injection of BiOI QDs (500 µg mL^−1^, 200 µL). Another parts of mice were sacrificed and the major organs or tissues were collected at the time of 1, 3, 5, 7, 14, and 28 day after intratumoral injection of BiOI QDs (2 mg mL^−1^, 50 µL).

## Conflict of Interest

The authors declare no conflict of interest.

## Supporting information

Supporting Information
