# Supplementary material for: Ultrasmall BiOI Quantum Dots with Efficient Renal Clearance for Enhanced Radiotherapy of Cancer
Source: Adv Sci (Weinh). 2020 Jan 16;7(6):1902561. doi: 10.1002/advs.201902561 (PMC7080545; doi:10.1002/advs.201902561)
Supplement: Supplementary file 1 — Supporting Information [file ADVS-7-1902561-s001.pdf]

Copyright WILEY-VCH Verlag GmbH & Co. KGaA, 69469 Weinheim, Germany, 2020.

ADVANCED SCIENCE
Open Access

Supporting Information

for *Adv. Sci.,* DOI: 10.1002/advs.201902561

Ultrasmall BiOI Quantum Dots with Efficient Renal Clearance for Enhanced Radiotherapy of Cancer

Xin Wang, Zhao Guo, Chenyang Zhang, Shuang Zhu, Lele Li, Zhanjun Gu, and Yuliang Zhao*

Copyright WILEY-VCH Verlag GmbH & Co. KGaA, 69469 Weinheim, Germany, 2019.

Supporting Information

Ultrasmall BiOI Quantum Dots with Efficient Renal Clearance for Enhanced Radiotherapy of Cancer

Xin Wang#, Zhao Guo#, Chenyang Zhang, Shuang Zhu, Lele Li, Zhanjun Gu, Yuliang Zhao*

Dr. X. Wang, Dr. Z. Guo, Dr. C. Zhang, S. Zhu, Prof. Z. Gu
CAS Key Laboratory for Biomedical Effects of Nanomaterials and Nanosafety, Institute of High Energy Physics, Chinese Academy of Sciences, Beijing, 100049, China
E-mail: zjgu@ihep.ac.cn

Dr. X. Wang, Dr. Z. Guo, Dr. C. Zhang, Prof. Z. Gu, Prof. Y. Zhao
College of Materials Science and Optoelectronic Technology, University of Chinese Academy of Sciences, Beijing 100049, China

Prof. L. Li, Prof. Y. Zhao
CAS Center for Excellence in Nanoscience, National Center for Nanoscience and Technology of China, Chinese Academy of Sciences, Beijing, 100190, China

[#]These authors contributed equally to this work.

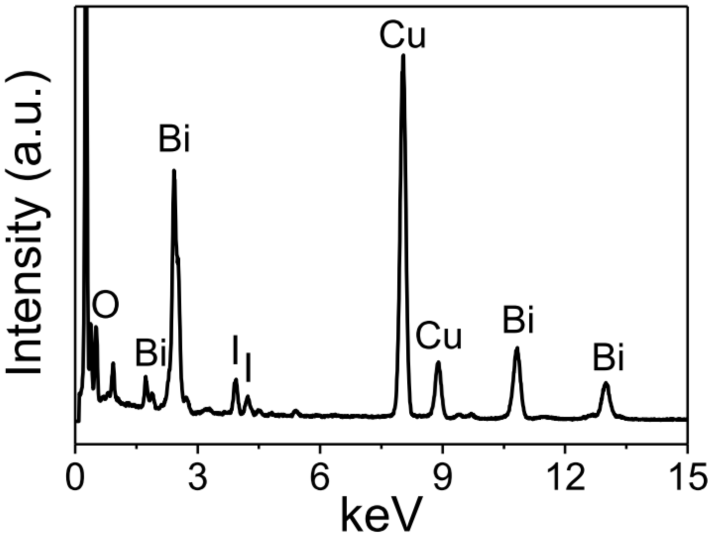

Figure S1. Energy-dispersive X-ray spectroscopy of BiOI QDs.

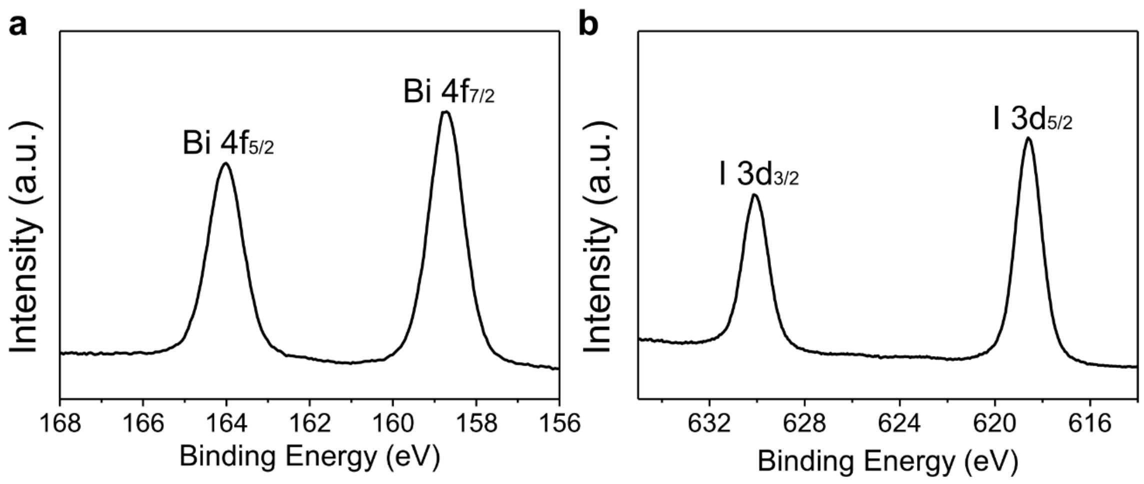

Figure S2. The high-resolution XPS spectra of (a) Bi 4f and (b) I 3d in BiOI QDs.

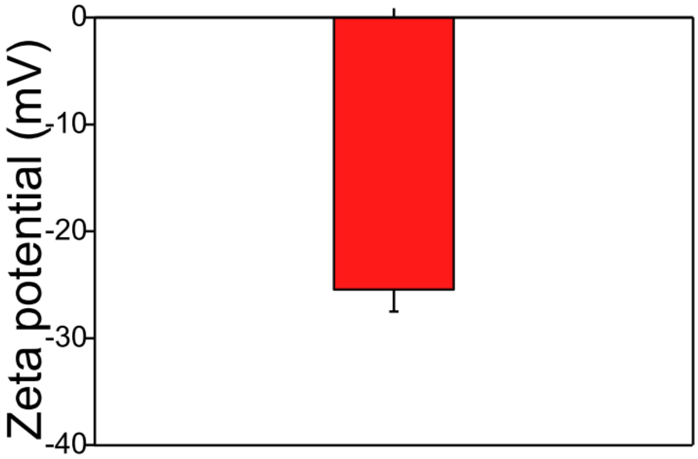

Figure S3. Zeta potential of as-prepared BiOI QDs.

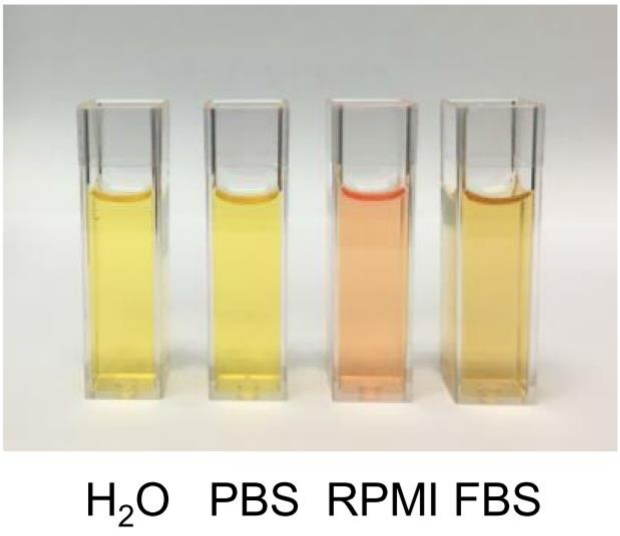

Figure S4. Photograph of BiOI QDs dispersing in various physical solutions after 24 h.

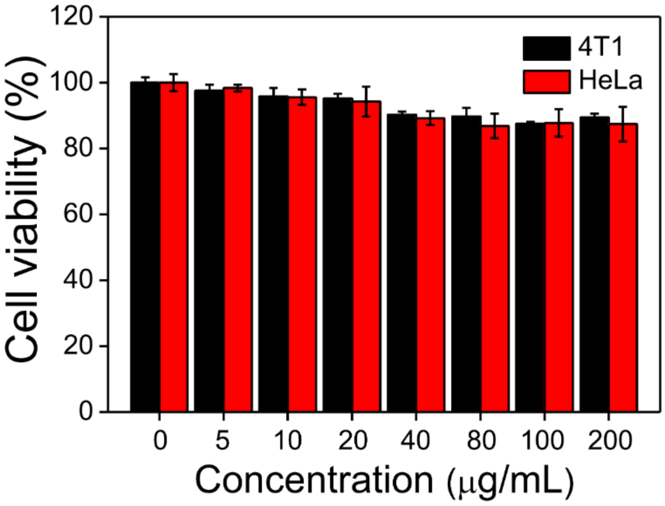

Figure S5. Cell viabilities of 4T1 cells and HeLa cells incubated with BiOI QDs of different concentrations for 24 h.

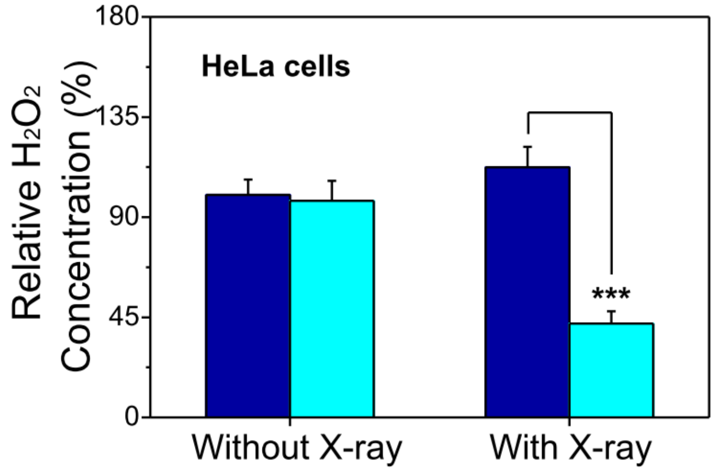

Figure S6. Detection of H_2O_2 concentration in HeLa cells after various treatments. P values were calculated by the Student's t test: ***$p < 0.001$.

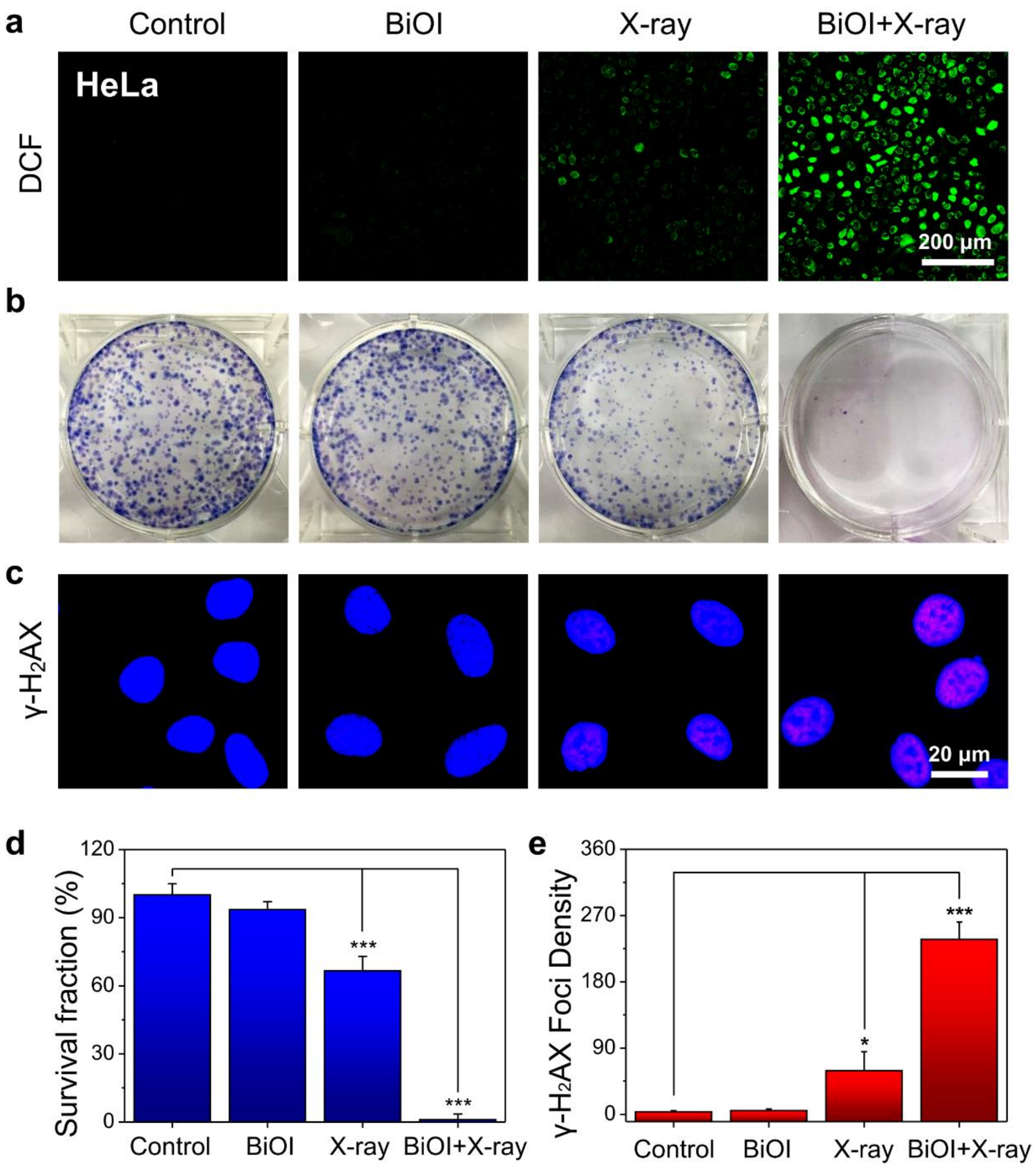

Figure S7. Enhanced radiotherapy of BiOI QDs in vitro on HeLa cells. a) ROS detection in HeLa cells. b) Colony formation after different treatments. c) Confocal images of DNA damage evaluated by γ-H_2AX. d) Survival fraction of each group from colony assay. e) Normalized γ-H_2AX fluorescence spot number after different treatments. P values were calculated by the Student's t test: *$p < 0.05$, ***$p < 0.001$.

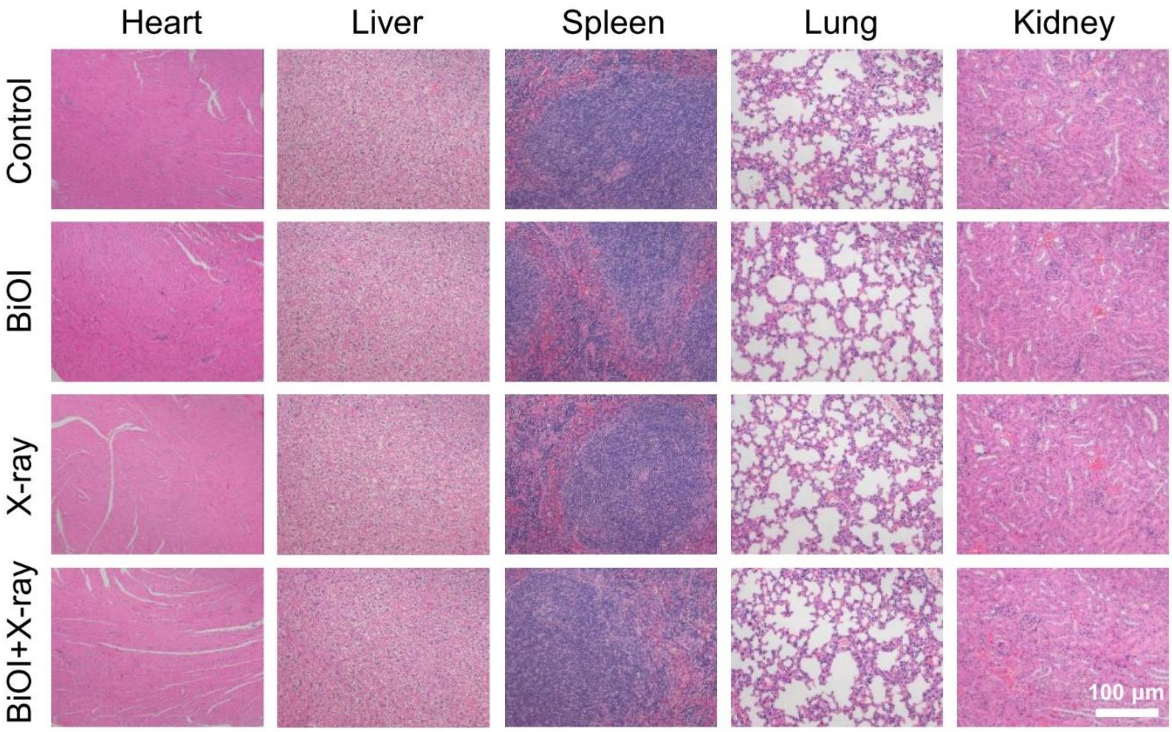

Figure S8. Representative H&E stained images of major organs from 4T1-tumor-bearing mice at the end of different treatments.

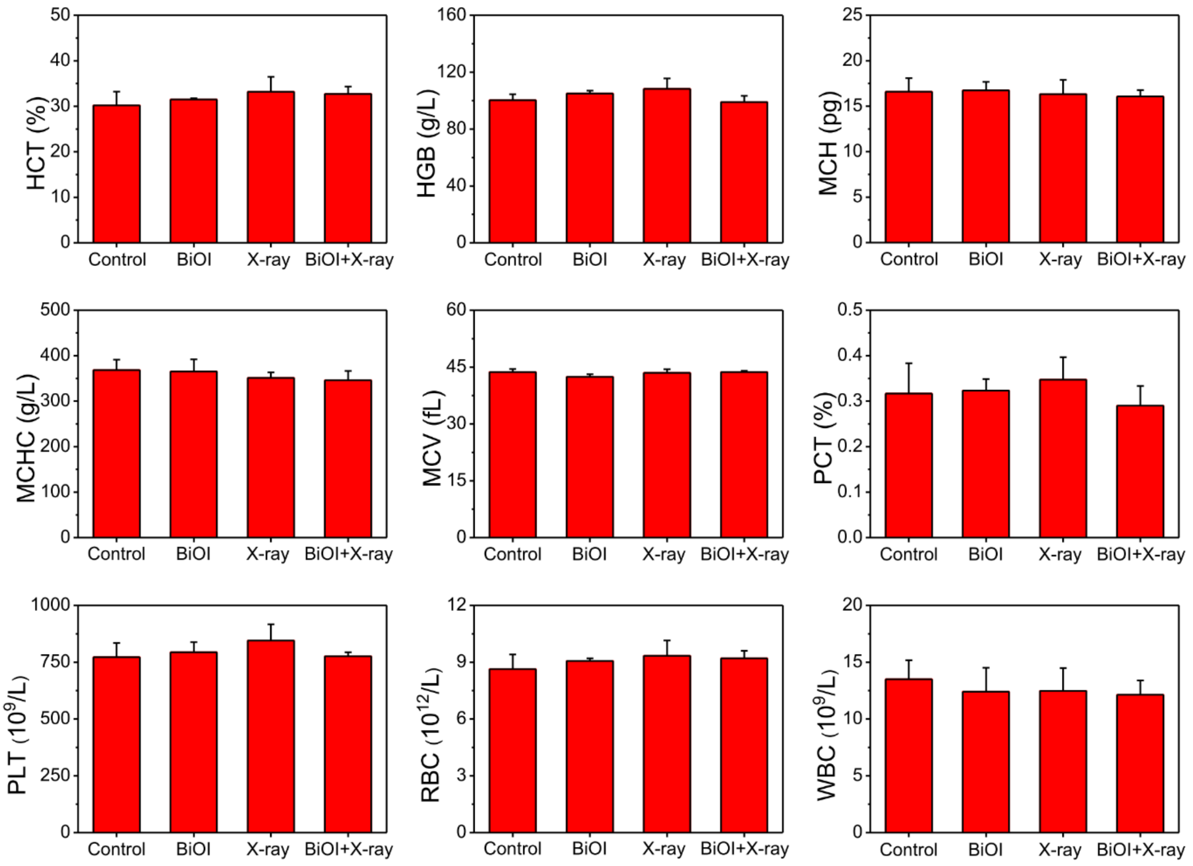

Figure S9. Blood hematology analyses of 4T1-tumor-bearing mice at the end of different treatments.

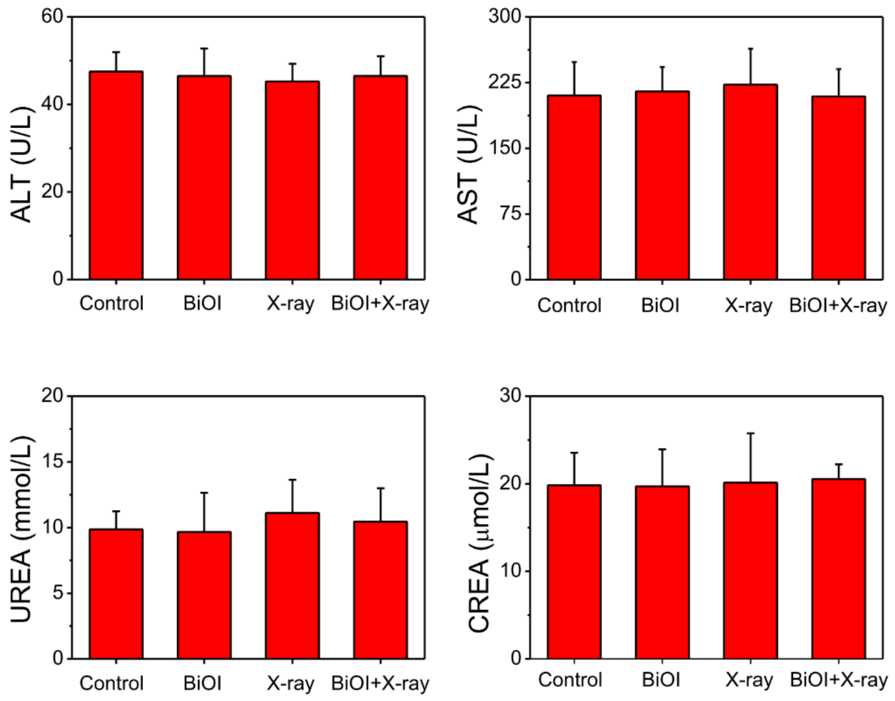

Figure S10. Blood biochemistry analyses of 4T1-tumor-bearing mice at the end of different treatments.

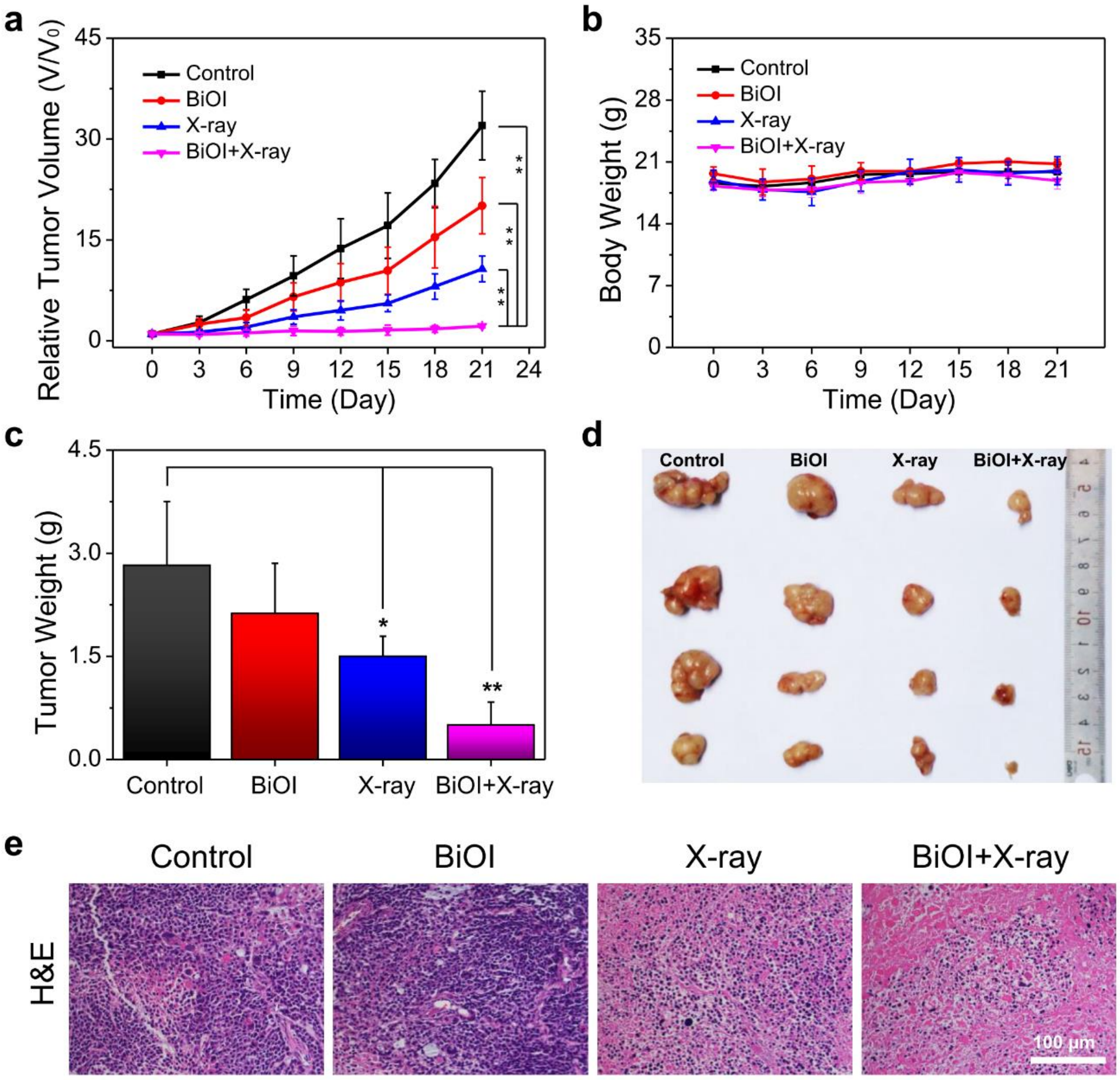

Figure S11. BiOI QDs sensitized radiotherapy in HeLa-tumor-bearing nude mice. a) Relative tumor volume profile of different groups: Control, BiOI, X-ray, BiOI + X-ray. b) Body weight of HeLa tumor bearing mice after treatments. c) Weights of excised tumors at 21st day after treatments. d) Photograph of excised tumors in each group at 21st day after treatments. e) Tumor images with H&E staining after treatments. P values were calculated by the Student's t test: *$p < 0.05$, **$p < 0.01$.

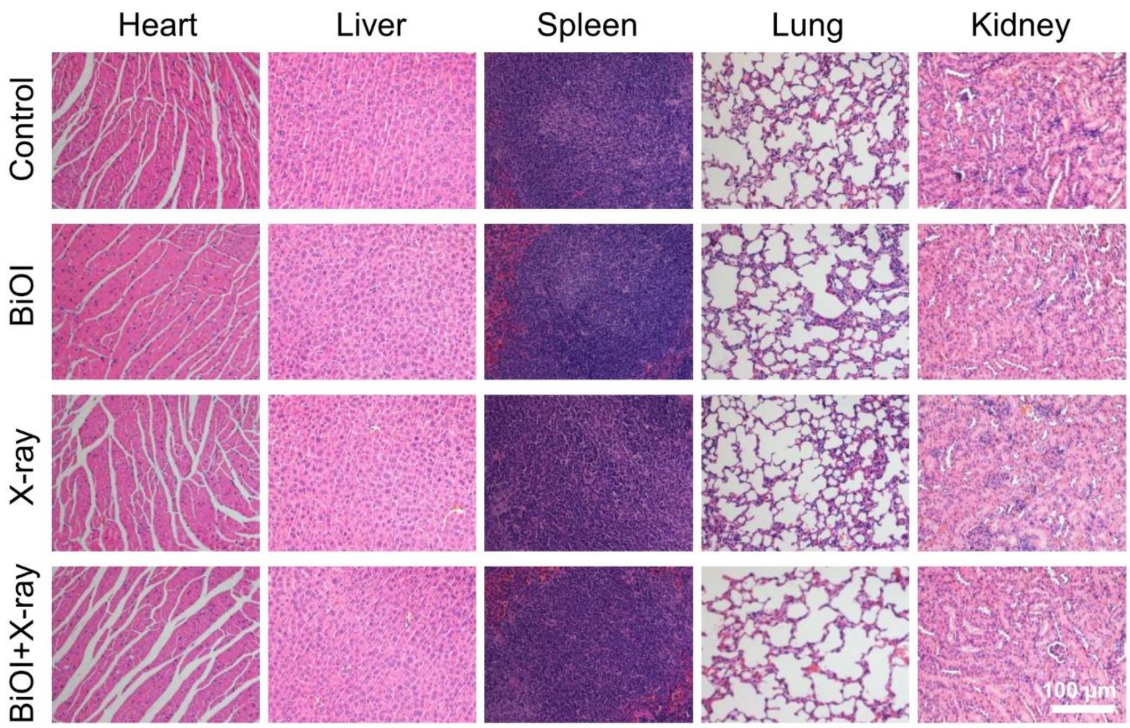

Figure S12. Representative H&E stained images of major organs from HeLa-tumor-bearing nude mice at the end of different treatments.

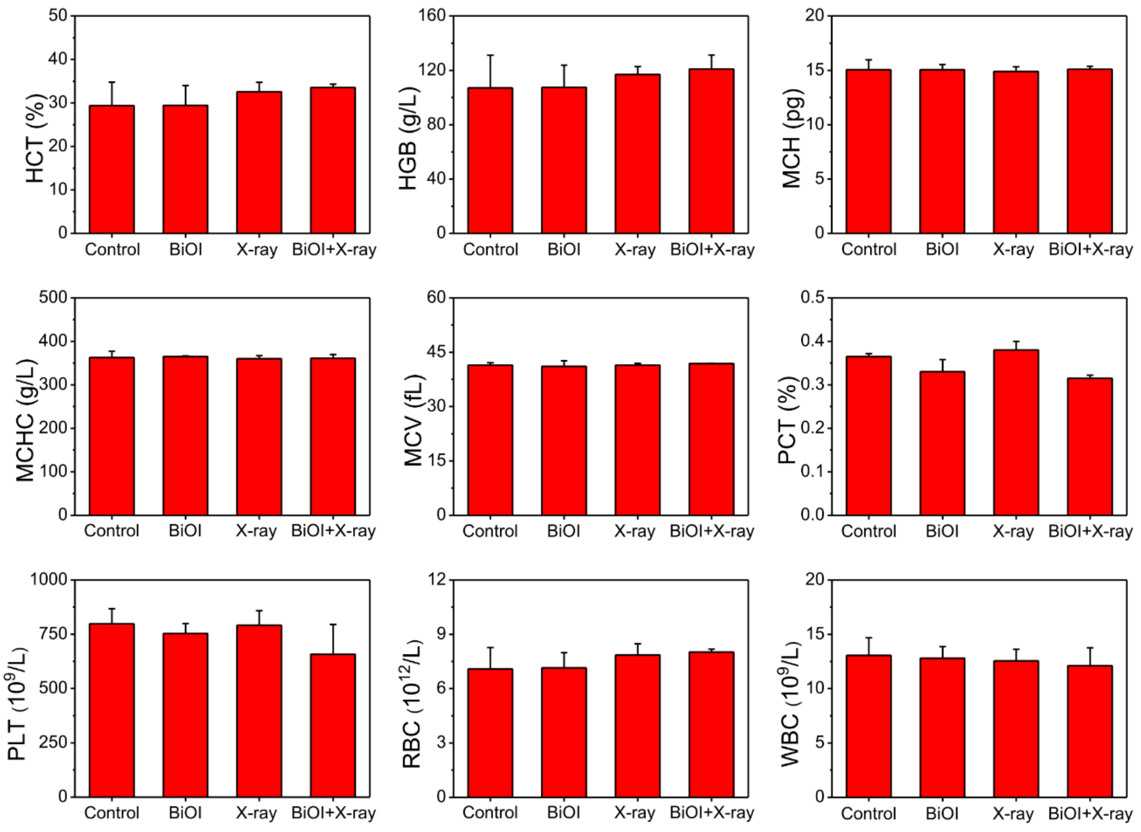

Figure S13. Blood hematology analyses of HeLa-tumor-bearing nude mice at the end of different treatments.

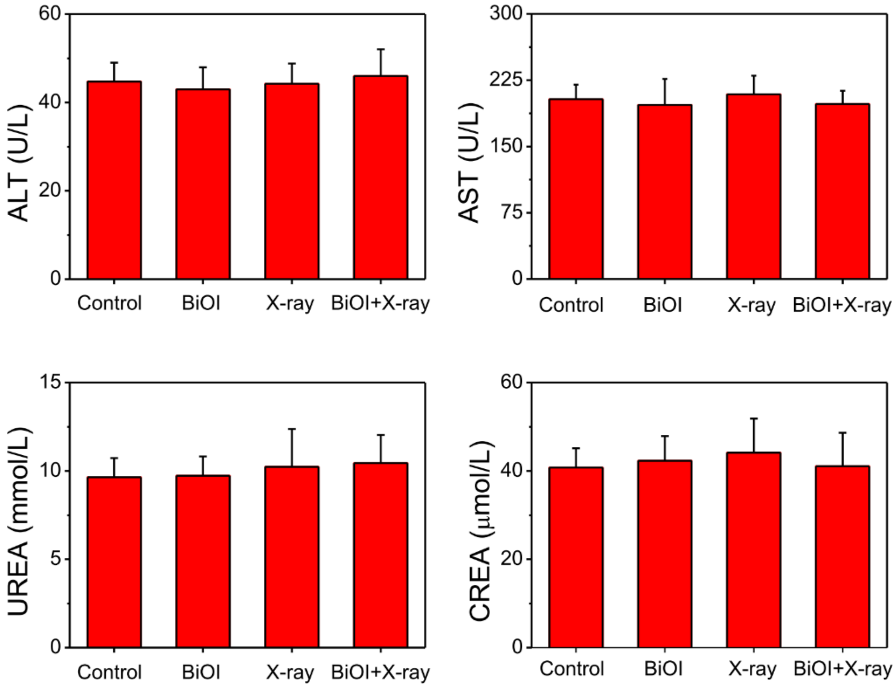

Figure S14. Blood biochemistry analyses of HeLa-tumor-bearing nude mice at the end of different treatments.
